# Unveiling the submerged secrets: bumblebee queens' resilience to flooding

**DOI:** 10.1098/rsbl.2023.0609

**Published:** 2024-04-17

**Authors:** Sabrina Rondeau, Nigel E. Raine

**Affiliations:** ^1^ School of Environmental Sciences, University of Guelph, Guelph, Ontario, Canada; ^2^ Department of Biology, University of Ottawa, Ottawa, Ontario, Canada

**Keywords:** diapause, bumblebees, inundation, flooding, climate change, hibernation

## Abstract

In a previous study, an experimental oversight led to the accumulation of water filling a container housing diapausing bumblebee queens. Surprisingly, after draining the water, queens were found to be alive. This observation raises a compelling question: can bumblebee queens endure periods of inundation while overwintering underground? To address this question, we conducted an experiment using 143 common eastern bumblebee (*Bombus impatiens*) queens placed in soil-filled tubes and subjected to artificially induced diapause in a refrigerated unit for 7 days. Tap water was then added to the tubes and queens (*n* = 21 per treatment) were either maintained underwater using a plunger-like apparatus or left to float naturally on the water’s surface for varying durations (8 h, 24 h or 7 days) while remaining in overwintering conditions. Seventeen queens served as controls. After the submersion period, queens were removed from water, transferred to new tubes with soil and kept in cold storage for eight weeks. Overall, queen survival remained consistently high (89.5 ± 6.4%) across all treatments and did not differ among submersion regimes and durations. These results demonstrate the remarkable ability of diapausing *B. impatiens* queens to withstand submersion under water for up to one week, indicating their adaptations to survive periods of flooding in the wild.

## 1. Introduction

In temperate regions, living organisms face the critical challenge of enduring adverse conditions during inhospitable periods, such as winter or dry seasons. To survive these challenging periods, many organisms rely on essential adaptations to synchronize their life cycle with seasonal changes [1]. In insects, one of the most common strategies to avoid the harsh cold conditions of winter is diapause, a state characterized by developmental dormancy [2,3].

Winter diapause occurs at various life stages, depending on the species, and involves a state of arrested or strongly delayed growth and reproduction [2,4]. In eusocial hymenopterans that form short-lived colonies, diapause is caste-dependent, with nest foundresses typically diapausing as mated adults [5,6]. For instance, in bumblebees (*Bombus* spp.), newly emerged gynes (unmated queens) are produced by the colony at the end of the summer and undergo mating before overwintering (diapausing) underground for 6–9 months [7,8]. These queens are the only individuals that survive the winter, while the workers and males perish before the winter months. While allowing the queens to escape the cold, underground overwintering means that diapausing queens are exposed to various stressors such as parasites [8,9], mould [10,11], soil contaminants [12–14] and flooding [15].

In the face of climate change, it is necessary to pay increased attention to how inhospitable climatic conditions affect living organisms. This includes the need to investigate potential adaptations and resilience mechanisms in response to anticipated temperature changes and the escalation of extreme climate events, such as droughts and floods [16,17]. In wild bees, some deleterious effects of warmer winter temperatures on diapausing individuals [18–20], including bumblebee queens [21], have already been reported. However, there is a dearth of knowledge regarding the potential impacts of flooding on subterranean diapausing species [15,22,23]. Given the global declines in bumblebee populations [24–27] and their vital role as pollinators, investigations into this taxon have become even more imperative. The response of bumblebee queens to environmental stressors is paramount considering that the success of a colony hinges entirely on the queen’s survival and her capacity to establish a new colony in the subsequent season.

In a previous study, an experimental oversight led to the inadvertent accumulation of water in containers housing diapausing bumblebee (*Bombus impatiens*) queens under controlled conditions [11]. Surprisingly, upon removing the water, the queens were found to be alive. Building on this intriguing observation, we conducted a study to determine whether bumblebee queens can endure periods of flooding while overwintering underground. Thus far, this line of research has remained entirely unexplored, even within literature specifically dedicated to bumblebee resilience to climate change [16]. Indeed, while submersion tolerance is rather common in some insect taxa, no study, to our knowledge has ever investigated the potential scenario of diapausing bumblebee queens withstanding submersion.

## 2. Methods

### 2.1. Queen rearing and diapause initiation

The common eastern bumblebee (*B. impatiens*) was chosen as a study organism owing to its commercial availability and widespread distribution and abundance in eastern North America [28]. Four queenright *B. impatiens* colonies were sourced from Biobest Canada Ltd (Leamington, Ontario, Canada) in January 2023 and kept in complete darkness in a bee-rearing room at 25°C and 60% relative humidity (RH). A red light was used for colony manipulations. Colonies were given ad libitum access to sugar solution (BIOGLUC®) and fresh frozen honeybee-collected pollen obtained from a local beekeeper. Newly emerged unmated queens (also referred to as gynes) were removed from their parental colonies upon eclosion and housed communally in aerated plastic containers (16 × 16 × 7.5 cm) with nest mates of the same age and colony, with a maximum of 10 gynes per cage. Separation from parental colonies, where males may be present, is essential to prevent queen mating (and inbreeding) and ensuring accurate tracking of gyne age at diapause onset, two factors that have been identified as potentially influencing survival during diapause [29,30]. These gynes were given unlimited access to fresh frozen pollen and 50% (w/v) sucrose solution (replaced every other day) until diapause onset (i.e. initiation of cold storage).

Queens were placed in cold storage when they reached an age range of 9–15 days, aligning with the optimal timeframe for entering diapause [29]. A total of 143 unmated queens were weighed and individually placed into 50 ml Falcon tubes, each containing 15 g of autoclaved topsoil (loamy sand: 80% sand, 15% silt, 5% clay) rewetted to 20% moisture content using deionized water. This soil was collected at a field site where bumblebees were previously observed digging hibernacula and was added to the tubes to enhance the ecological relevance of the study. The tube lids were pierced with ventilation holes and the tubes were kept in a dark refrigerated unit at 4°C and 55% RH for 7 days before the application of different submersion treatments (acclimation period).

It should be noted that while photoperiod and temperature typically dictate the onset and termination of diapause in insects [31], the specific mechanisms governing these phases in wild bumblebee queens remain unclear [32]. Nevertheless, subjecting laboratory-reared *B. impatiens* queens to cold storage has consistently been used to successfully induce diapause in the laboratory [29,30,33]. Unmated queens were used to avoid introducing potential confounding effects of mating variation on the experiment. Although diverging from natural conditions, prior research has shown that queens successfully enter and survive diapause in laboratory settings, irrespective of their mating status [29,34,35], although unmated queens could be slightly more likely to survive diapause [34,36].

### 2.2. Submersion treatments

**Figure 1.**
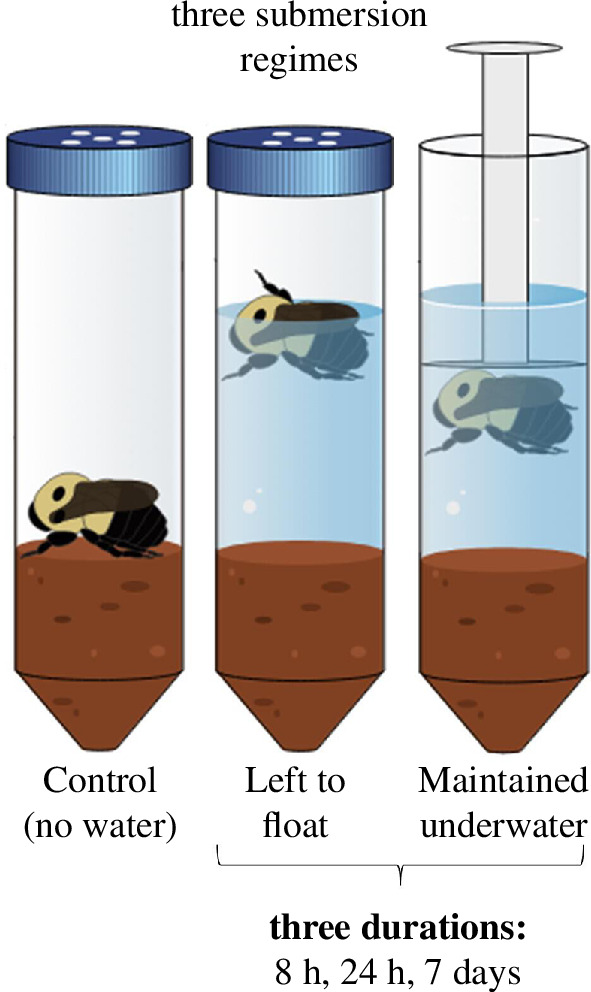
Schematic representation of experimental design. Bumblebee graphic modified from the original by Jeremy Hemberger.

Survival was assessed before treatment application by exposing queens to room temperature and ambient light for 5 min. Queens that displayed distinct movement (e.g. leg twitching or abdominal movements) were considered alive [37,38] and were subjected to one of three submersion treatments for varying durations, using a fractional–factorial design (figure 1). Seventeen queens served as controls without any submersion treatment and were put back into the refrigerated unit for 7 days. Cool tap water (4°C, 20 ml) was added to the remaining tubes and queens (*n* = 21 per treatment) were either maintained under water using a plunger-like apparatus or left to float naturally on the water’s surface for varying durations (8 h, 24 h or 7 days) while remaining in overwintering conditions within the refrigerated unit (figure 1). The different submersion regimes and durations simulated different flooding scenarios, ranging from light (short-duration floods of 1 day or less) to moderate (complete inundation of dry land of 7 days) inundation events [39]. More specifically, leaving queens to float on the water’s surface simulated a scenario of high groundwater levels caused by heavy rainfall, snow melt or floods, where water would accumulate in the hibernaculum without completely filling it. Conversely, a scenario of complete land inundation would result in queens being entirely submerged.

### 2.3. Queen survival

After the submersion period, queens were removed from water, transferred to new tubes with soil (as described above) and kept in cold storage until the end of the experiment. Queens from the control group were also transferred to new tubes after 7 days. Queen survival was assessed one, four and eight weeks from the day the submersion treatments were applied. Queens marked as dead were left at room temperature for 2 h to confirm this status.

### 2.4. Analysis

Statistical analyses were performed in R v.4.3.1. We used generalized linear models with a binomial error distribution to assess the impact of submersion treatments on queen survival. Separate models were fitted for each assessment date (i.e. one, four and eight weeks post-treatment). In all models, the submersion treatment (i.e. the combination of submersion regime and duration) was used as a fixed effect, with parental colony and queen weight included as covariates. Missing values for the weight of 14 queens from one parental colony were replaced by the average weight of all queens in the analysis. We tested whether submersion affected queen survival by performing likelihood ratio tests (*α* = 0.05) of models with and without the treatment variable. Model diagnostics were performed using diagnostic plots (quantile-quantile plot and residuals plot) generated from the DHARMa package.

## 3. Results

**Figure 2.**
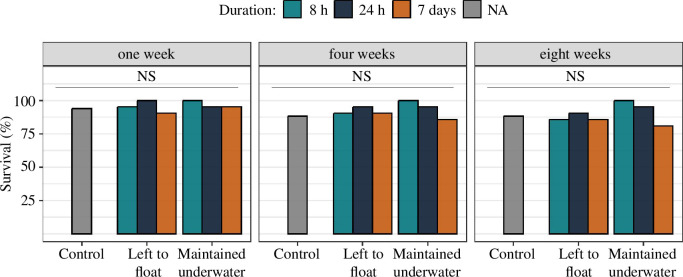
Survival of diapausing bumblebee queens subjected to varying submersion regimes and durations. Twenty-one queens were used per treatment, with 17 queens serving as controls. Survival was assessed one, four and eight weeks from the day the submersion treatments were applied.

Survival of diapausing queens remained consistently high across all experimental groups up to eight weeks post-treatment (mean ± s.d.: 89.5 ± 6.4%). Notably, 81% (17 out of 21) of the queens that were subjected to the most intense submersion regime and duration (i.e. maintained under water for 7 days) were still alive after eight weeks of artificial overwintering, compared to 88% (15 out of 17) for the control group (table 1; figure 2).

**Table 1.** Queen survival rates across treatment groups at one, four and eight weeks post-treatment, along with likelihood ratio test (LRT) output for group comparisons.

	survival (%)							LRT output		
	control	left to float			maintained under water			treatment	queen weight	parental colony
	---	8 h	24 h	7 d	8 h	24 h	7 d			
one week	94.1	95.2	100	90.5	100	95.2	95.2	χ² = 4.86,*p* = 0.562	χ² = 5.21,*p* = 0.022	χ² = 3.95,*p* = 0.267
four weeks	88.2	90.5	95.2	90.5	100	95.2	85.7	χ² = 5.52,*p* = 0.479	χ² = 17.52,*p* <0.001	χ² = 3.36,*p* = 0.339
eight weeks	88.2	85.7	90.5	85.7	100	95.2	81.0	χ² = 7.55,*p* = 0.273	χ² = 11.35,*p* <0.001	χ² = 0.61,*p* = 0.895

The difference in model fit between models with and without the submersion treatment variable was not significant at any assessment timepoint (eight weeks post-treatment: χ² = 7.55; *p* = 0.273), indicating that submersion did not affect queen survival. Survival was found to be significantly influenced by queen weight, with a higher probability of survival observed in heavier queens (table 1).

4. Discussion.

Flooding poses an unpredictable challenge for subterranean species, a phenomenon that has been little studied in bees that nest or overwinter underground [40]. Our study unequivocally demonstrates the remarkable ability of diapausing *B. impatiens* queens to withstand submersion under water for up to one week, indicating their adaptations to survive land flooding in the wild.

Many terrestrial insects spend part, or all, of their lives in soil and are well adapted to this flood-prone habitat [41–43]. Among the key features facilitating life underground, these insects showcase a remarkable array of adaptations that enable them to cope with varying degrees of hypoxia [44–46], a key challenge posed by flooding. These adaptations encompass a shift from aerobic to anaerobic metabolic pathways, the capacity to swiftly decrease basal metabolic rates, behavioural changes and compensatory adjustments in tracheal morphology [42,44,45].

Submersion tolerance is particularly prevalent among insects inhabiting floodplains, with the most flood-resistant species employing egg diapause as a strategy to cope with flooding [41,43]. Flood-tolerant diapausing eggs are common among collembolans, grasshoppers, planthoppers and leafhoppers [43,47]. In certain species, pupae exhibit greater flood tolerance than larvae [42], with the diapausing pre-pupae of specific ground-nesting bee species capable of enduring extended periods of up to six months under water [23,48]. By contrast, few examples of flooding tolerance are available among species undergoing diapause as adults [41,43], making bumblebee queens a rare case among documented flood-tolerant species. Indeed, previous studies have shown that most insects overwintering as adults (e.g. many ground beetles) are unable to tolerate winter submersion in floodplains and instead migrate to sheltered areas to evade unfavourable conditions [43].

A range of adaptations may contribute to bumblebee queens’ resilience to flooding. For instance, many diapausing insects, including bumblebee queens, employ discontinuous gas exchange, keeping spiracles closed for extended periods with only brief intermittent bursts of gas exchange [49,50]. This respiratory pattern primarily serves to reduce the risk of desiccation and exposure to environmental challenges, such as hypoxia, but may also reduce water entry through spiracles during submersion [42,51,52]. Even when spiracles are open, water entry is probably limited by the small diameter of spiracle openings and the highly hydrophobic cuticle of insects [42]. Furthermore, cuticular gas exchange and/or the presence of trapped air bubbles that act as physical gills may be sufficient to meet the low oxygen requirements associated with diapause [44,45,51].

The fact that submersion tolerance is typically observed in insect taxa residing in periodically submerged habitats makes the resilience of bumblebee queens to flooding even more surprising [41]. This is because existing literature suggests a tendency for overwintering bumblebee queens to avoid flood-prone soils. Indeed, these queens are most often observed overwintering in sloping, sandy and well-drained ground [7,8,14,53,54], probably to evade the challenges of extreme humidity and flooding. Yet, these features may offer limited protection in the face of rising groundwater that could flood the hibernaculum and our results indicate that, in the event of a severe inundation, bumblebee queens are equipped to survive submersion independently of overwintering site characteristics.

While our results show that diapausing bumblebee queens can survive periods of complete submergence of up to 7 days, information regarding longer-term exposure and potential long-term effects is currently lacking and requires further investigation. Among bees, prolonged flooding has been shown to induce high mortality in populations of two ground-nesting species, *Andrena vaga* [22] and *Lasioglossum zephyrum* [55], presumably by depriving nesting larvae or (pre)pupae of oxygen. In another study, extended spring flooding also appeared to reduce bee abundance and species richness, hinting at a mortality effect on bee populations [15]. Similar responses have been observed across taxa, where an increase in flooding frequency and duration typically leads to reduced abundance and biomass of soil macrofauna [41]. Physiological impairment after flooding is also common, even in flood-resistant insect species [41].

Findings from this research pave the way for a wide array of new research questions. Among the many, key future directions include:

- documenting the respiratory conditions within bumblebee queen hibernacula and exploring the mechanisms and metabolic implications underlying their resilience to flooding;
- documenting the long-term effects of diverse flooding scenarios, including instances of multiple freeze-thaw cycles, on the survival of bumblebee queens and their subsequent overall colony fitness;
- assessing the combined impacts of flooding and other stressors (e.g. pesticide exposure, temperature fluctuations, diseases and parasites, fungal growth) on diapausing bumblebee queens;
- comparing the resilience to flooding between commercial and wild *B. impatiens* queens and among different bumblebee species;
- assessing bumblebee queen resilience to flooding under field conditions;
- investigating potential delays in emergence from diapause, as suggested for ground-nesting bees [23], and exploring the associated phenological implications of flooding on bumblebee queens; and
- exploring whether adult‐overwintering solitary ground-nesting bees can similarly withstand submersion.

Conditions increasing mortality during diapause can have devastating effects on bumblebee populations by reducing the number of new queens establishing colonies in the spring. Despite the decline observed in many bumblebee species, *B. impatiens* stands out as relatively resilient [25,26,56]. Exploring whether its ability to withstand flooding plays a role in these discrepancies constitutes a key avenue for investigation. In the face of global bumblebee declines, understanding the mechanisms and impact of diapause disruptions holds important ecological implications. This is especially pertinent within the context of ongoing climate change, where the escalation of extreme events, such as flooding, is anticipated.

## Data Availability

The data and R code supporting this study are publicly available on Dryad [57].
